# Novel Protocol for Persister Cells Isolation

**DOI:** 10.1371/journal.pone.0088660

**Published:** 2014-02-21

**Authors:** Silvia J. Cañas-Duarte, Silvia Restrepo, Juan Manuel Pedraza

**Affiliations:** 1 Department of Biological Sciences, Universidad de los Andes, Bogota, Colombia; 2 Department of Physics, Universidad de los Andes, Bogota, Colombia; Tel Aviv University, Israel

## Abstract

Bacterial persistence, where a fraction of a population presents a transient resistance to bactericidal substances, has great medical importance due to its relation with the appearance of antibiotic resistances and untreatable bacterial chronic infections. The mechanisms behind this phenomenon remain largely unknown in spite of recent advances, in great part because of the difficulty in isolating the very small fraction of the population that is in this state at any given time. Current protocols for persister isolation have resulted in possible biases because of the induction of this state by the protocol itself. Here we present a novel protocol that allows rapid isolation of persister cells both from exponential and stationary phase. Moreover, it is capable of differentiating between type I and type II persister cells, which should allow the field to move beyond its current state of studying only one type. While this protocol prompts a revision of many of the current results, it should greatly facilitate further advances in the field.

## Introduction

Antibiotic resistance is a well-known phenomenon whereby microbial populations acquire mutations that render them immune to certain antibiotics. Bacterial persistence is a different phenomenon [1] where small fractions of clonal bacterial populations can survive lethal doses of antimicrobial agents [1]–[4] while the population as a whole remains susceptible. This happens through a partially understood mechanism where individual cells can stochastically enter into a transient state characterized by arrested or reduced growth [5], [6]. This phenomenon appears to be widely spread among prokaryotic microorganisms and from its first reports in the late 1944 in *Staphylococcus aureus* [7], to the identification of high persistence strains in *Escherichia coli* in the late 80’s [8], [9], persistence occurrence has been reported in several other bacterial strains, including human pathogens such as *Pseudomonas aeruginosa* and the etiologic agent of tuberculosis *Mycobacterium tuberculosis* [3], [10]–[12]. Persister cells tolerance to antibiotic agents and their presence in biofilms has been associated with chronic recalcitrant bacterial infections [3], [13]. The study of bacterial persistence is therefore of great importance to public health.

In spite of its medical relevance, the biochemical and evolutionary underpinnings of persistence have only been partially elucidated. Persister cells are known to be generated stochastically at low frequencies in populations of *E. coli* [5] in constant conditions. This might constitute a strategy for population survival in changing environments [14], [15], possibly acting as an “insurance policy” in case of a catastrophic event [2], [14]. Several recent studies in *E. coli* have proposed that both Toxin-Antitoxin (TA) loci and stress response mechanisms may be involved in the generation of persister cells [3], [4], [6], [15]–[19], but although evidence supporting the above mentioned hypothesis had been recently discovered in *E. coli*, the full mechanism behind persister cell generation remains largely elusive [3], [6]. The transient nature of persistence, the low frequencies of persister cells generation and its relationship with preexisting phenotypic heterogeneity in bacterial populations [2], [5] have been the main limitations for the study of persistence.

To circumvent the problem of low frequencies, high persistence mutants of *E. coli* (*hip* mutants) have been the focus of study. Among them, two different persister types have been identified [5], [9], [20]. Although both types of persisters are not susceptible to antibiotics and preexist in bacterial populations, they differ in that type I persisters are non-growing cells that seem to be generated upon passage to stationary phase whereas type II persisters are slow-growing cells that generate continuously during exponential growth in a fashion that seems to depend on the population size [5]. The above mentioned differences among type I and type II persister cells indicate that different mechanisms may govern their generation, and therefore studying both persister types separately might contribute to our understanding of the general mechanism of persistence in bacteria. Although type I and type II persisters are present in wild type *E. coli* populations [5], their differentiation had only been achieved using Time-Lapse Single Cell Microscopy techniques. Because both types appear to be equally non susceptible to antibiotics [5], their isolation for biochemical analysis of only one type was impossible using traditional protocols for persister isolation. Furthermore, the relevant mutation has been isolated only in the case of type I high-persistence mutants.

Current isolation methods rely on the activity of antibiotic agents [3], [5], [15]–[19], [21] and require long exposition times. They are also bound to the specific killing kinetics of each antibiotic for normally growing cells, resulting in methods that are highly dependent on the physiological state of the cells [22]–[24]. Furthermore, prolonged exposure to antibiotic agents often activates stress response mechanisms, which have been recently related to persistence induction [1], [3], [17], [21], [25]. This means that the methods used to isolate persister cells are also inducing their generation, which precludes experiments determining how the persister fraction changes under different stimuli or studying the transcriptomics of persisters separately from their response to antibiotics.

In the present study, we were able to develop a novel method for persister isolation, based on a combination of alkaline and enzymatic lysis that targets the cell membrane. Our protocol for persister cells isolation is not only much faster than traditional methods and hence less likely to induce a stable stress response mechanism but also allows the differentiation and isolation of the two persister types that coexist in *E. coli* populations. Furthermore our protocol is also independent of the physiological state and the size of the bacterial population, and works in different bacterial species like *P. fluorescens* and even in the gram-positive bacteria *S. aureus* (Supporting information File S1). This novel protocol for persister cells isolation would significantly facilitate future studies of persistence in several bacterial strains, and thus improve our understanding of the mechanisms underlying this phenomenon.

## Materials and Methods

### Bacterial Strains and Growth Conditions

The bacterial strains used in this work and their relevant characteristics are listed in Table 1. Experiments were conducted on Luria-Bertani (LB) medium at 37°C and 200 rpm unless otherwise specified. For the analysis of antibiotic based persisters isolation protocols, LB broth was supplemented with Ampicillin (100 ug/mL) or Ofloxacin (10 ug/mL).

**Table 1:** *Escherichia coli* strains used in this study.

Strains	Relevant features	Source or reference
TH1268	MG1655 zde264::Tn10	Korch et al, 2003
TH1269	MG1655 *hipA7* zde264::Tn10	Korch et al, 2003
DS1	*hipQ*	Wolfson et al, 1990

### Standardization of Persister Frequencies of E. coli Strains TH1268, TH1269 and DS1 using Traditional Isolation Methods based on Antibiotics

Commonly used protocols for persister cells isolation were used as described [5], [17], [18] with the *Escherichia coli* K12 TH1268, TH1269 (*hipA7*) and DS1 (*hipQ*) strains in both exponential and stationary growth phases.

For the exponential phase experiments, 300 mL of fresh LB broth was inoculated with 10 uL of an ON culture. Experiments were conducted when the culture reached OD = 0.4 (more than 6 hours of incubation). Stationary phase analyses were performed using 1∶1 and 1∶100 dilutions of an ON culture.

Killing curves were constructed by taking 1 mL aliquots at different times, serially diluting each aliquot and plating on LB plates for determination of cfu counts.

### Novel Protocol for Persister Cells Isolation

Our protocol isolates persister cells by rapidly killing normally growing cells using a mixture of lytic solutions. Exponential phase and stationary phase cultures were grown as described above. For persister isolation one aliquot of 1 mL was taken from the culture in the desired growth phase and 200 µL of the lysis solution (Sigma, Miniprep Kit) was added in a 15 mL falcon. The mixture was then homogenized using vortex for 10 seconds and then incubated at room temperature for 10 minutes.

After completion of the 10 min. incubation, 200 µL of the enzymatic lysis solution (45 mg,48539 units/mg, of Lysozyme (Sigma) in 1 mL of TE buffer) were added to the mixture and gently homogenized by inverting the capped falcon. Finally the preparation was incubated for 15 minutes at 37°C at 200 rpm. Each aliquot was serially diluted and plated on LB plates for determination of persister cell frequencies.

### Type I and Type II Persister Cells Differentiation

The protocol can be used to selectively kill normally growing cells, leaving type I and type II persisters, or to selectively kill both normally growing cells and type II persisters leaving only type I persister cells. Type I persisters were isolated by using the lytic protocol as described above adding 500 µL of both the osmotic lysis solution (Sigma, Miniprep kit) and the Lysozyme lytic solution instead of 200 µL.

### Validation of Persister Cells Isolation using Time Lapse Microscopy

Persister cells were isolated as described and then concentrated using centrifugation. The lytic mixture was washed out and replaced by fresh LB medium. A 10 µL aliquot was then observed using DIC microscopy with a special growth chamber that allowed single cell monitoring during 10 hours at 37°C.

## Results and Discussion

### Comparison between Commonly Implemented Persister Cells Isolation Methods

Current methods for persister isolation use different antibiotics, lengths of treatment and dilutions of the cultures [3]–[5], [15], [16], [19], [21], [26]. They generate different persister fractions depending on treatment and on the growth phase. While differences due to growth phase could provide valuable insights on the molecular mechanisms underpinning the persistence state [22]–[24], differences due to treatment could be due to the killing kinetics of each antibiotic or to stress induced persistence induction. They could also mean that persistence is antibiotic-specific [6], [15], [17], [21], though this seems unlikely given previous observations of their preexistence [5].

**Figure 1:**
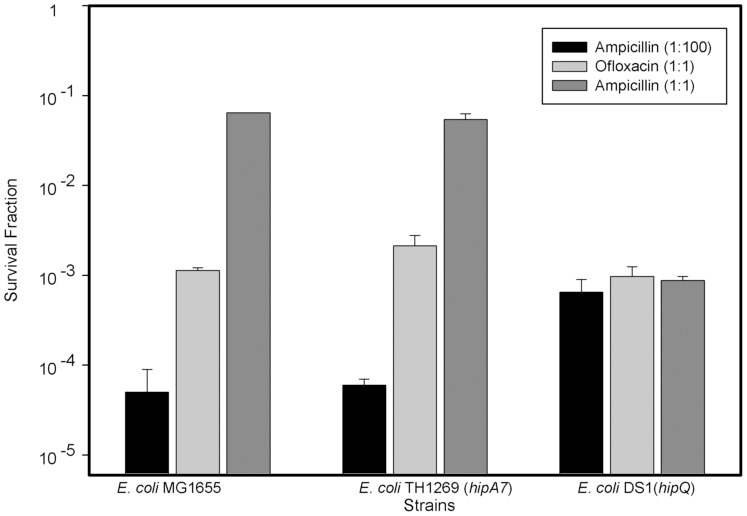
Comparison between Ampicillin and Ofloxacin based persister cells isolation methods in 3 *E. coli* strains. Using different antibiotics and methodologies for persister isolation generated markedly different survival fractions. Ampicillin and Ofloxacin were tested using exponentially growing cultures without dilution (1∶1) at an OD of 0.5. As a reference in this figure, we used the results of ampicillin treatment with an initial dilution of the culture (1∶100) reported by Balaban et al. in 2004 [5]. Error bars indicate the standard deviation (n = 3), except for the Ampicillin (1∶100) treatment in which the error bars indicate the range reported by Balaban for exponentially growing cultures.

**Figure 2:**
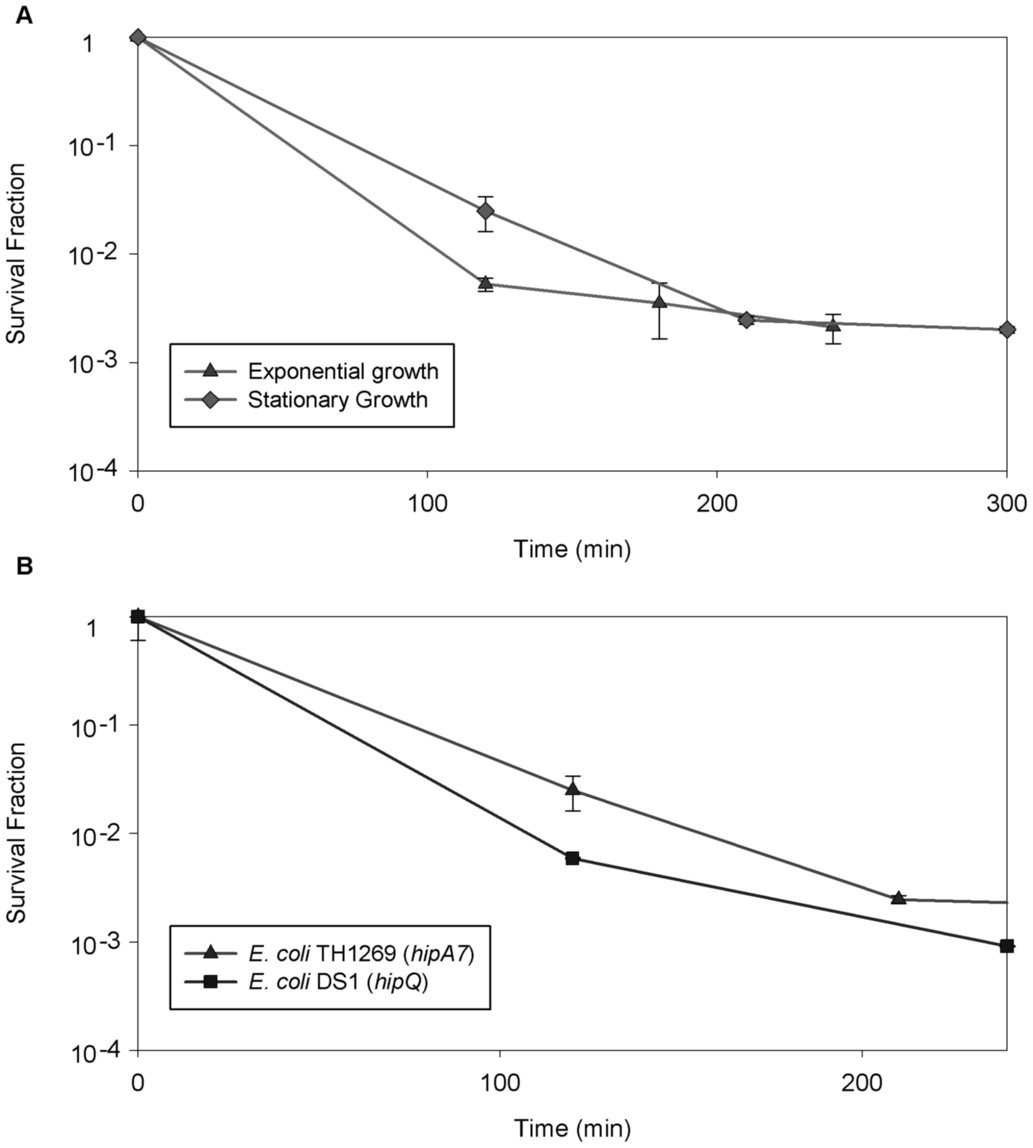
Persister cells isolation in *E. coli* strains using Ofloxacin. The killing kinetics of Ofloxacin showed differences in stabilization time when comparing its activity in stationary and exponential growth phase, requiring almost double time when acting in stationary phase cells, even in the same *E. coli* strain (A). Survival fractions from stationary phase cultures of two different *E. coli* strains also exhibited markedly different stabilization times, even in the same physiological state (B). Error bars indicate the standard deviation (n = 3).

To assess these protocols, we compared those based on Ampicillin [5] and Ofloxacin [17], using the different lengths of treatment and initial dilutions of the samples (Figure 1). As mentioned, the fraction of surviving cells varied strongly amongst the tested isolation protocols, and these differences were strain dependent. We also measured the killing kinetics of the above mentioned antibiotics in different physiological states and in different *E. coli* strains (Figure 2). These treatments required a long incubation of at least 3 hours to reach stabilization and we observed differences in stabilization times amongst physiological states (Figure 2A) and between different strains (Figure 2B).

### Development and Standardization of the Lytic Protocol for Persister Cell Isolation

Any treatment that lasts more than 45 minutes risks the stable activation of a stress response mechanism and therefore the induction of persistency. An improved method for persister cells isolation should thus be significantly faster than traditional methods. Additionally, it should stably maintain persister cells after the isolation to allow further studies on the isolated persister cells. Finally, an optimal protocol should be as insensitive as possible to the population size, the physiological state of the culture and the bacterial strain.

**Figure 3:**
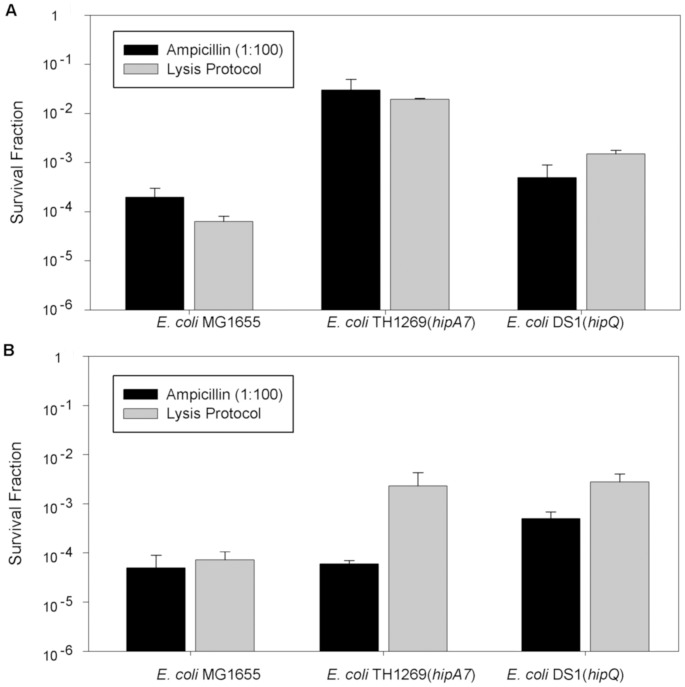
Standardization of the lysis protocol for persister cells isolation. For the characterization of our protocol for persister cells isolation we calculated persistence frequencies (survival fractions) in both stationary (A) and exponential (B) growth of three different *E. coli* strains, using 200 uL of each lytic solution for the exponentially growing cultures and 500 uL for the stationary cultures. We compared the results obtained using our protocol with those reported by Balaban et al. in 2004 (Ampicillin, 1∶100) for all the tested strains. Error bars indicate the standard deviation (n = 3), except for the Ampicillin (1∶100) treatment in which the error bars indicate the range reported by Balaban for cultures of the above mentioned strains.

**Figure 4:**
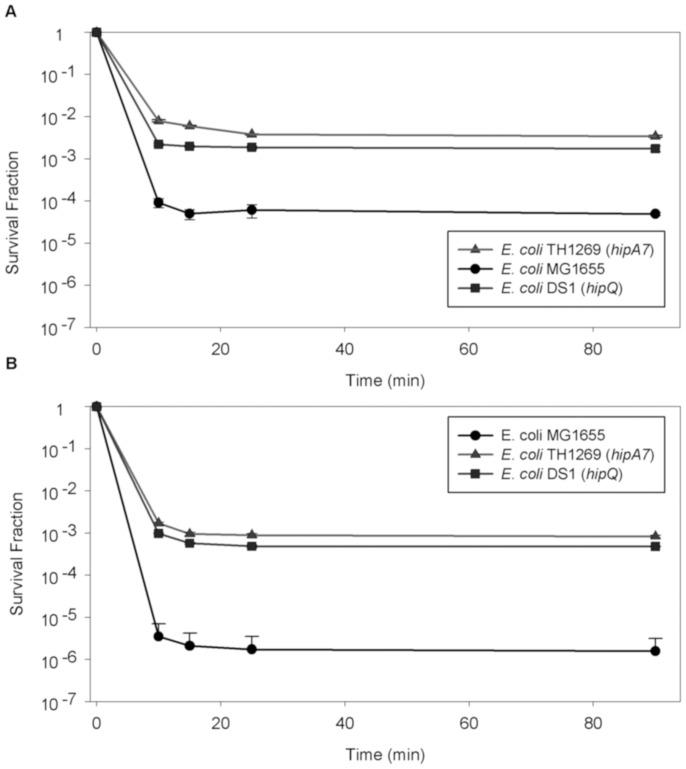
Persister cells isolation: Killing curves. For all the tested strains our protocol reached stabilization shortly (15 minutes) after the addition of the enzymatic lytic solution and remained stable for more than 90 minutes in both exponentially (A) and stationary (B) growing cultures. For the exponentially growing cultures, 200 uL of each lytic solution was added to isolate persister cells as previously described, whereas for the stationary cultures we used 500 uL of each solution. Error bars indicate the standard deviation (n = 3).

We initially tested isolation cocktails with both higher concentrations and combination of several antibiotic agents, but found that using this strategy isolation was never achieved in less than 45 minutes. We then tested detergents such as Triton X and SDS both alone and combination with antibiotics to enhance their action, but this also failed to fulfill the desired characteristics (data not shown). In the end we discovered that lytic solutions were a viable option. After testing different lytic cocktails we found that a combined protocol, in which an osmotic lysis solution is added for 10 minutes, followed by an enzymatic lysis solution incubated for 15 minutes at 37 C, allowed us to stably isolate persister cells from both stationary and exponential growth phase in less than 25 minutes. We compared the results obtained with our novel protocol with the standards for persister frequencies reported by Balaban in 2004 in three *E. coli* strains (Figure 3) and observed that our protocol produced persistence frequencies similar to those originally reported [5] for stationary phase but with a notable difference for the *hipA7* strain in exponential phase. To further standardize our new protocol we performed killing curves with the same three *E. coli* strains (Figure 4) in which persister cell frequencies had been extensively characterized [5]. We confirmed that fast isolation is achieved with our protocol and also that persister cells isolated remained viable and available for further studies for more than 90 minutes. Furthermore, the protocol works with other bacterial species like *P. fluorescens* and *S. aureus* (Supporting Information File S1).

### Isolation and Differentiation between Type I and Type II Persisters

**Figure 5:**
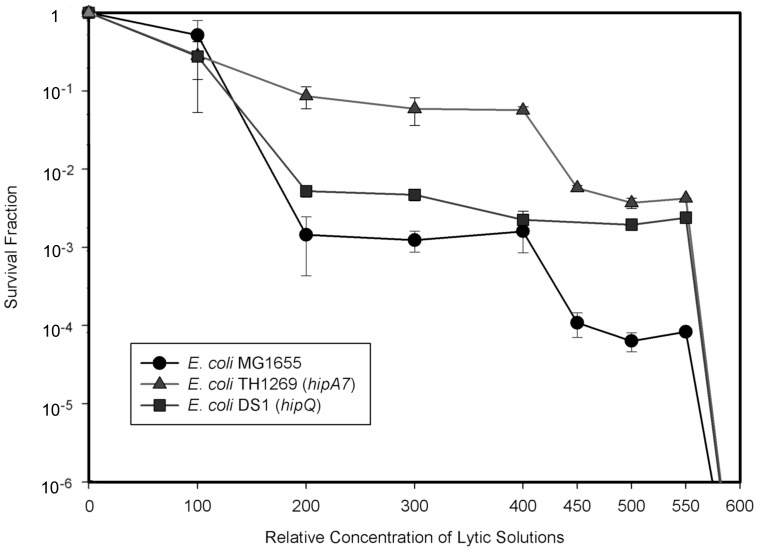
Differentiation between type I and type II persister cells from Stationary phase cultures. Using different working concentration of our lysis solutions we obtain two marked plateaus in the killing curves. The first one corresponds to a mixture of type I and type II persister cells, while the second one corresponds to type I persister cells exclusively. The fraction of type I cells in the first plateau depends on the fraction of type I and type II persisters in the population, which in turn depends on strain and growth conditions.

Type I and type II persister cells are equally non susceptible to antibiotic insults, and therefore previously used methods are unable to differentiate among them. So far the only differentiation between persister cell types was achieved using time lapse microscopy techniques and microfluidic devices by Balaban and collaborators [14]. To determine if our protocol could differentiate between type I and type II persisters, we tested a broad range of effective concentrations of our lytic protocol and we observed two marked plateaus in the resulting killing curves that correspond respectively to type I and type II persister cells together or to type I persisters alone (Figure 5). The fraction of type I cells in the first plateau depends on the fraction of type I and type II persisters in the population, which in turn depends on strain and growth conditions. Results are equivalent for exponentially growing cultures.

**Figure 6:**
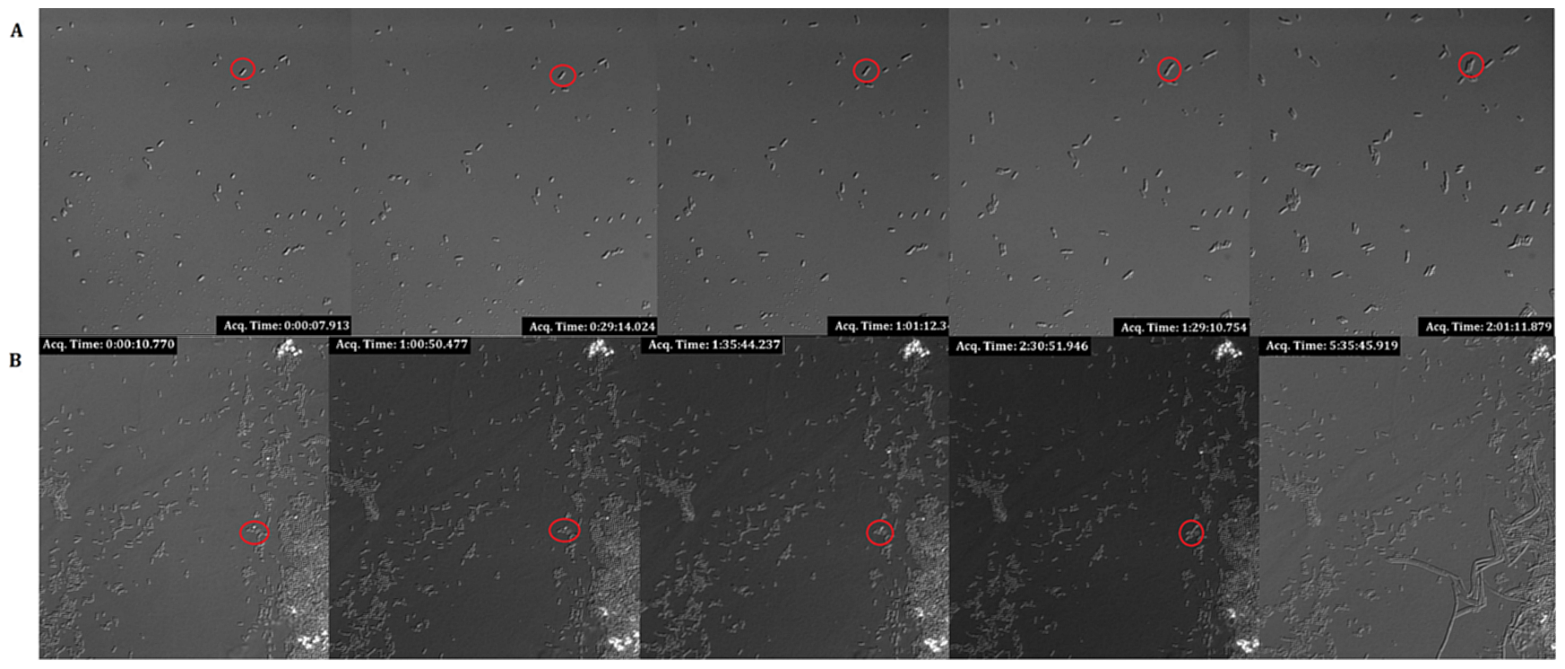
Time lapse microscopy validation of type I and type II persister cells isolation. Using DIC microscopy we assessed the performance of our novel protocol in persister cells isolation and for differentiating between type I and type II persister cells. A) Type I and II persister cells were isolated from a stationary phase culture of *E. coli* DS1. Cell division time was found to be on average 159 minutes +/30 minutes (n = 36) for the cells that were growing. B) A stationary phase culture of *hipA7* (TH1269) was treated with our protocol to differentially isolate type I persister cells. All cells were found to be non-growing whilst in the persistence state and after switching back to a normally growing state cell division occurred rapidly.

These results indicate that while both exponentially and stationary growing cells are equally susceptible to our lytic cocktail, there might be differences in the cell envelope between type I and type II persister cells that confers them differential tolerance to it. Using Time Lapse microscopy we validated that each of the isolated fractions with our lytic protocol behave as previously reported for both type I and type II persister cells [5]. Type I persisters exhibited arrested growth for an average of 3 hours after which rapid growth was resumed (Figure 6B) whilst type II persister cells presented slow growth, cell division occurring in average once every 159 minutes (Figure 6A). The full image sequences are provided as supporting information (File S2).

The advantages of our new protocol allowed us to isolate type II persisters during exponential growth for transcriptomic profiling. The complete results will be reported elsewhere, but they confirmed that none of the genes from S.O.S response, known to be activated by antibiotics, were induced during the implementation of our protocol for persisters isolation. The new protocol we present should allow many more studies of this type, allowing the separate study of the mechanisms of stochastic persistence induction and their interaction with stress response systems in both types of persistence.

## Supporting Information

File S1 **Supporting Information.** Includes information on the testing of the protocol in other strains and the statistical analysis of the differences between commonly implemented persister isolation protocols and the protocol presented here.
(DOCX)

File S2 **Microscopy images.** Isolation of type I and type II persister cells. As described in Figure 6, time-lapse microscopy was implemented to validate the isolation of type I and type II persisters using our protocol. Figures S2 to S7 are a sequence of images taken of the isolation of type I persisters from a stationary phase culture of *hipA7* (TH1269) whilst Figures S8 to S12 correspond to the isolation of type I and type II persister cells from an stationary culture of *E. coli* DS1 (*hipQ*).
(ZIP)
