# Supplementary material for: Novel Protocol for Persister Cells Isolation
Source: PLoS One. 2014 Feb 21;9(2):e88660. doi: 10.1371/journal.pone.0088660 (PMC3931647; doi:10.1371/journal.pone.0088660)
Supplement: File S1 — Supporting Information. Includes information on the testing of the protocol in other strains and the statistical analysis of the differences between commonly implemented persister isolation protocols and the protocol presented here. (DOCX) [file pone.0088660.s001.docx]

**Supporting information File S1**

**Statistical analysis of the differences between commonly implemented persister isolation protocols.**

As shown in Figure 1, we found strong differences between the results of three persister isolation protocols. Using the One-way ANOVA test with the statistical software Minitab 16, we analyzed the significance of the differences found. With a threshold p-value of 0.05 we found that the differences were significant for both the TH1269 (*hipA7*) and the MG1655 (WT) strain. For the *E. coli* DS1 strain, we found that the differences are not significant, as can be seen in the figure.

**Testing of the protocol in other strains**

We used the protocol on *P. fluorescens* and *S. aureus* and cultures in stationary phase We used 500μl of the lysis solution for *S. aureus* and a full sweep of concentrations for *P. fluorescens*. We obtain a persistent fraction of 2.03*10^-4^ for *S. aureus*. For *P. fluorescens* we obtained a single plateau in the killing curves (Fig. S1). Our results suggest that there is only one type of persister cells in the tested strain of *P. fluorescens.*





**Figure S1. Persister cells isolation in *P. fluorescens*.** To test if our protocol was suitable at similar concentrations for using in different bacterial species we assessed it with a stationary culture of *P. fluorescens* using different working concentrations of our lysis solutions, as done previously for *E. coli*.
