# Supplementary figures and images for: Novel Protocol for Persister Cells Isolation
Source: PLoS One. 2014 Feb 21;9(2):e88660. doi: 10.1371/journal.pone.0088660 (PMC3931647; doi:10.1371/journal.pone.0088660)

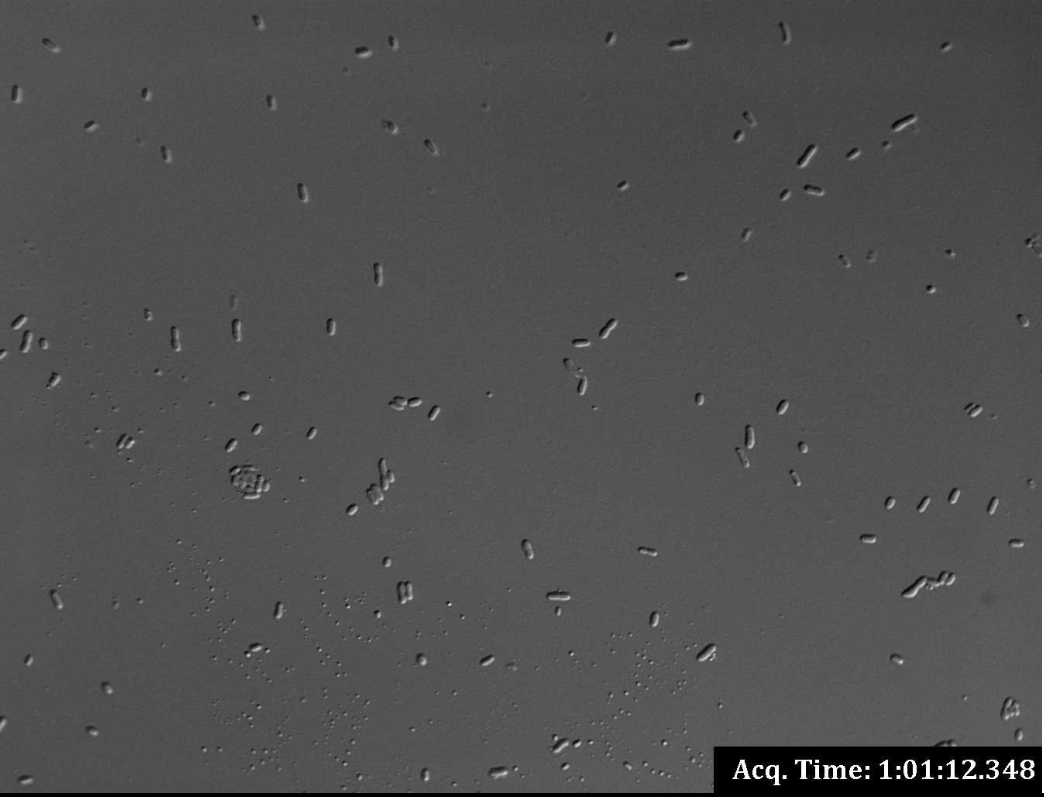

Supplement: File S2 — Microscopy images. Isolation of type I and type II persister cells. As described in Figure 6, time-lapse microscopy was implemented to validate the isolation of type I and type II persisters using our protocol. Figures S2 to S7 are a sequence of images taken of the isolation of type I persisters from a stationary phase culture of hipA7 (TH1269) whilst Figures S8 to S12 correspond to the isolation of type I and type II persister cells from an stationary culture of E. coli DS1 (hipQ). (ZIP) [file pone.0088660.s002.zip › PHOTOS/S10.tif]

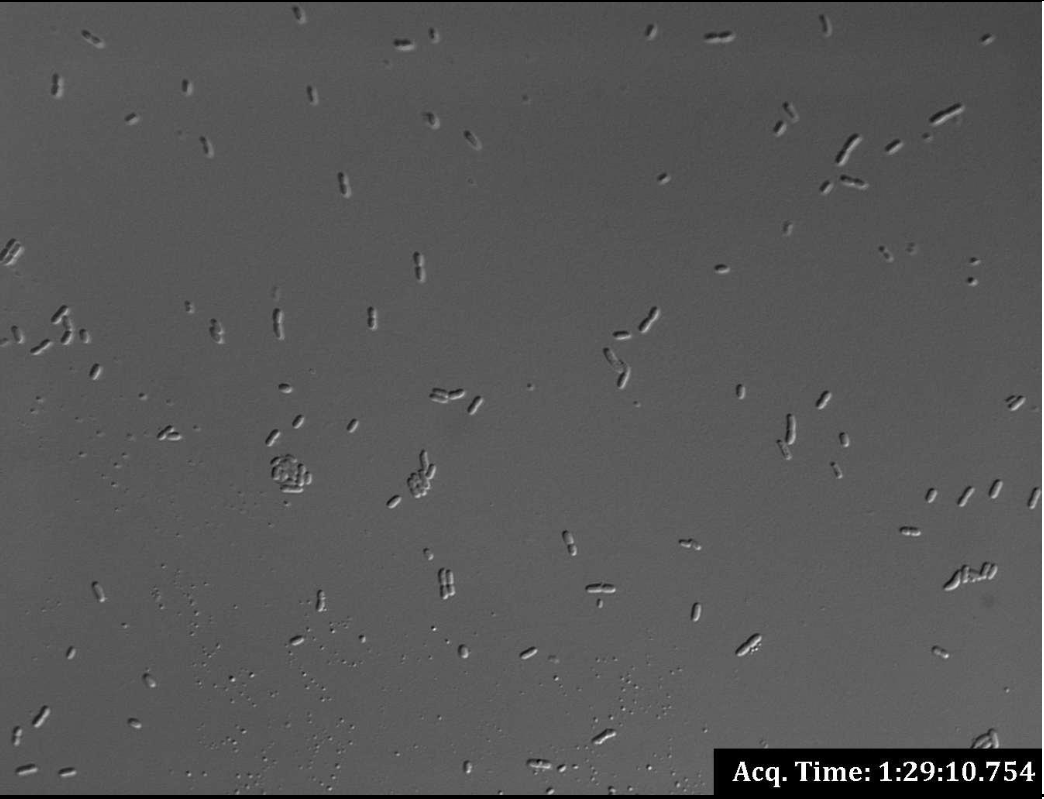

Supplement: File S2 — Microscopy images. Isolation of type I and type II persister cells. As described in Figure 6, time-lapse microscopy was implemented to validate the isolation of type I and type II persisters using our protocol. Figures S2 to S7 are a sequence of images taken of the isolation of type I persisters from a stationary phase culture of hipA7 (TH1269) whilst Figures S8 to S12 correspond to the isolation of type I and type II persister cells from an stationary culture of E. coli DS1 (hipQ). (ZIP) [file pone.0088660.s002.zip › PHOTOS/S11.tif]

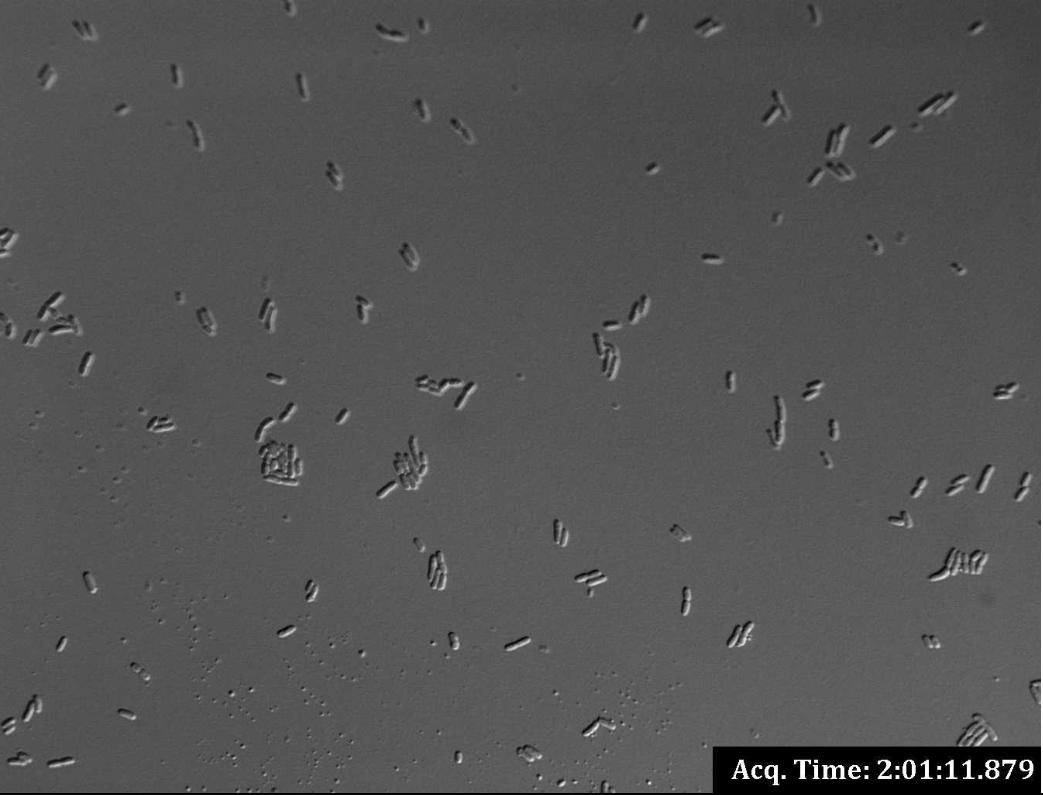

Supplement: File S2 — Microscopy images. Isolation of type I and type II persister cells. As described in Figure 6, time-lapse microscopy was implemented to validate the isolation of type I and type II persisters using our protocol. Figures S2 to S7 are a sequence of images taken of the isolation of type I persisters from a stationary phase culture of hipA7 (TH1269) whilst Figures S8 to S12 correspond to the isolation of type I and type II persister cells from an stationary culture of E. coli DS1 (hipQ). (ZIP) [file pone.0088660.s002.zip › PHOTOS/S12.tif]

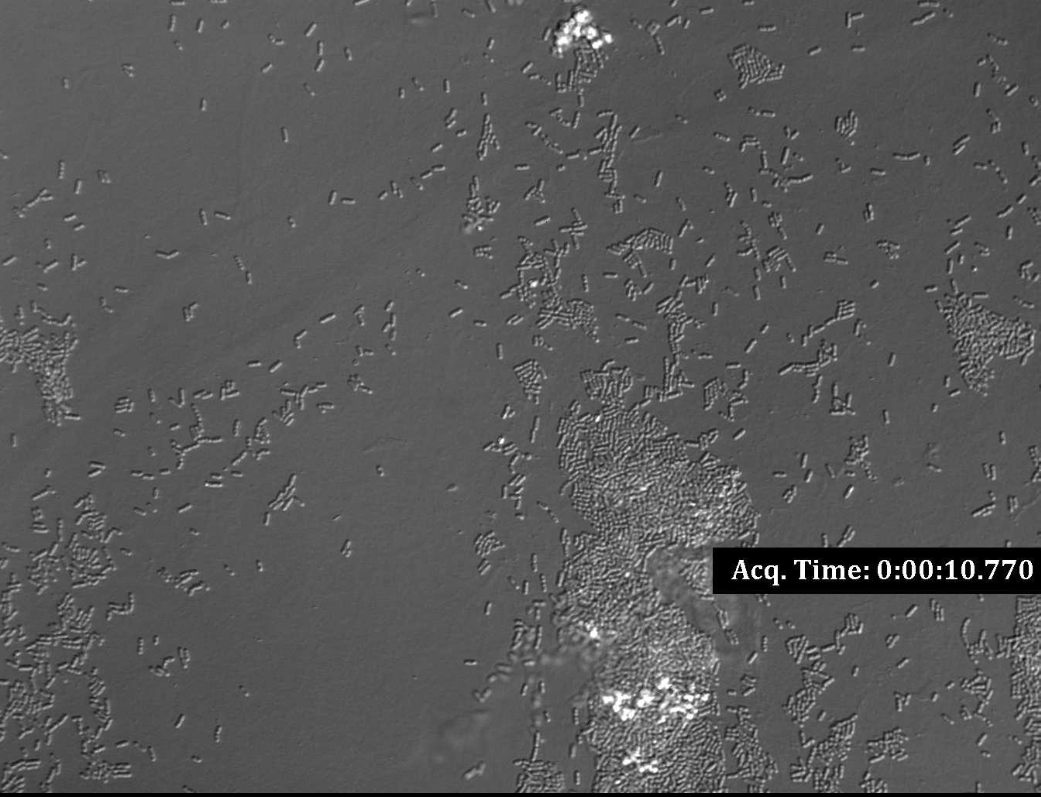

Supplement: File S2 — Microscopy images. Isolation of type I and type II persister cells. As described in Figure 6, time-lapse microscopy was implemented to validate the isolation of type I and type II persisters using our protocol. Figures S2 to S7 are a sequence of images taken of the isolation of type I persisters from a stationary phase culture of hipA7 (TH1269) whilst Figures S8 to S12 correspond to the isolation of type I and type II persister cells from an stationary culture of E. coli DS1 (hipQ). (ZIP) [file pone.0088660.s002.zip › PHOTOS/S2.tif]

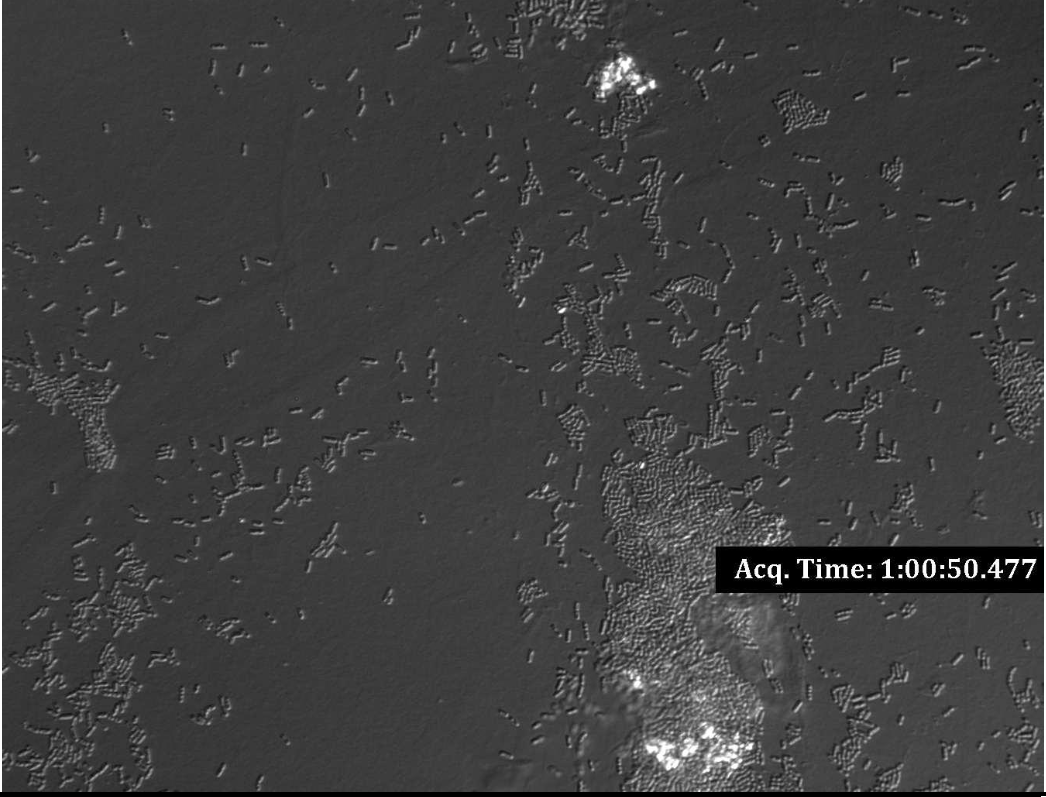

Supplement: File S2 — Microscopy images. Isolation of type I and type II persister cells. As described in Figure 6, time-lapse microscopy was implemented to validate the isolation of type I and type II persisters using our protocol. Figures S2 to S7 are a sequence of images taken of the isolation of type I persisters from a stationary phase culture of hipA7 (TH1269) whilst Figures S8 to S12 correspond to the isolation of type I and type II persister cells from an stationary culture of E. coli DS1 (hipQ). (ZIP) [file pone.0088660.s002.zip › PHOTOS/S3.tif]

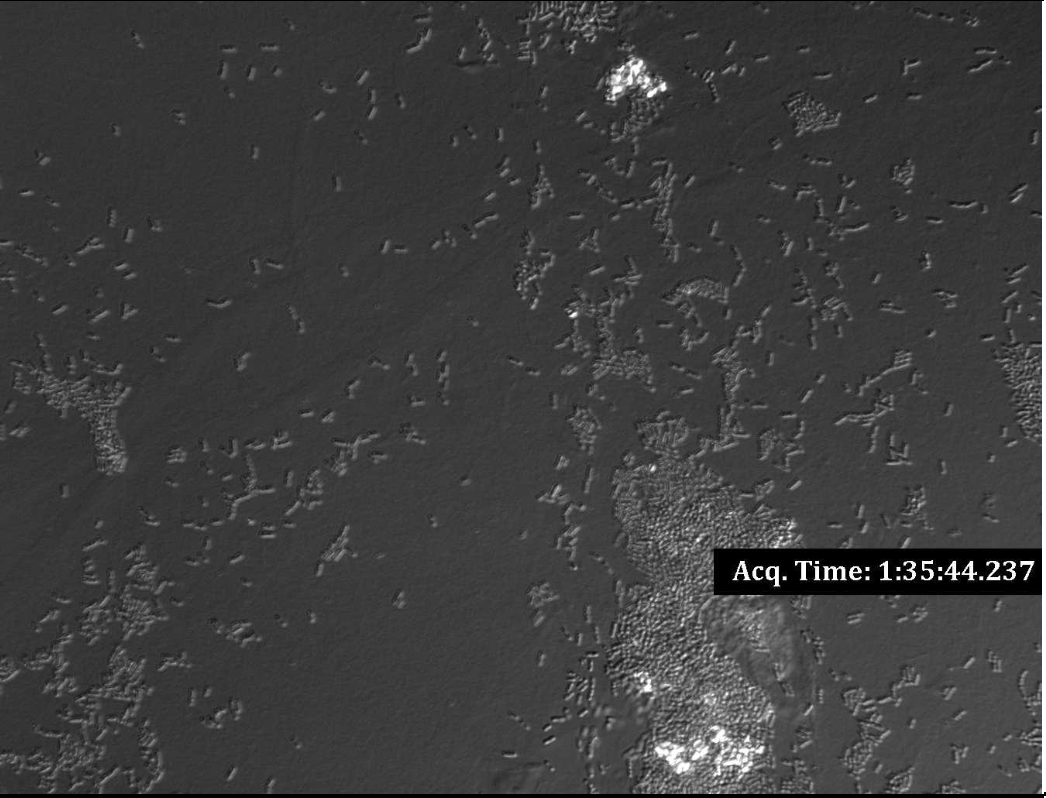

Supplement: File S2 — Microscopy images. Isolation of type I and type II persister cells. As described in Figure 6, time-lapse microscopy was implemented to validate the isolation of type I and type II persisters using our protocol. Figures S2 to S7 are a sequence of images taken of the isolation of type I persisters from a stationary phase culture of hipA7 (TH1269) whilst Figures S8 to S12 correspond to the isolation of type I and type II persister cells from an stationary culture of E. coli DS1 (hipQ). (ZIP) [file pone.0088660.s002.zip › PHOTOS/S4.tif]

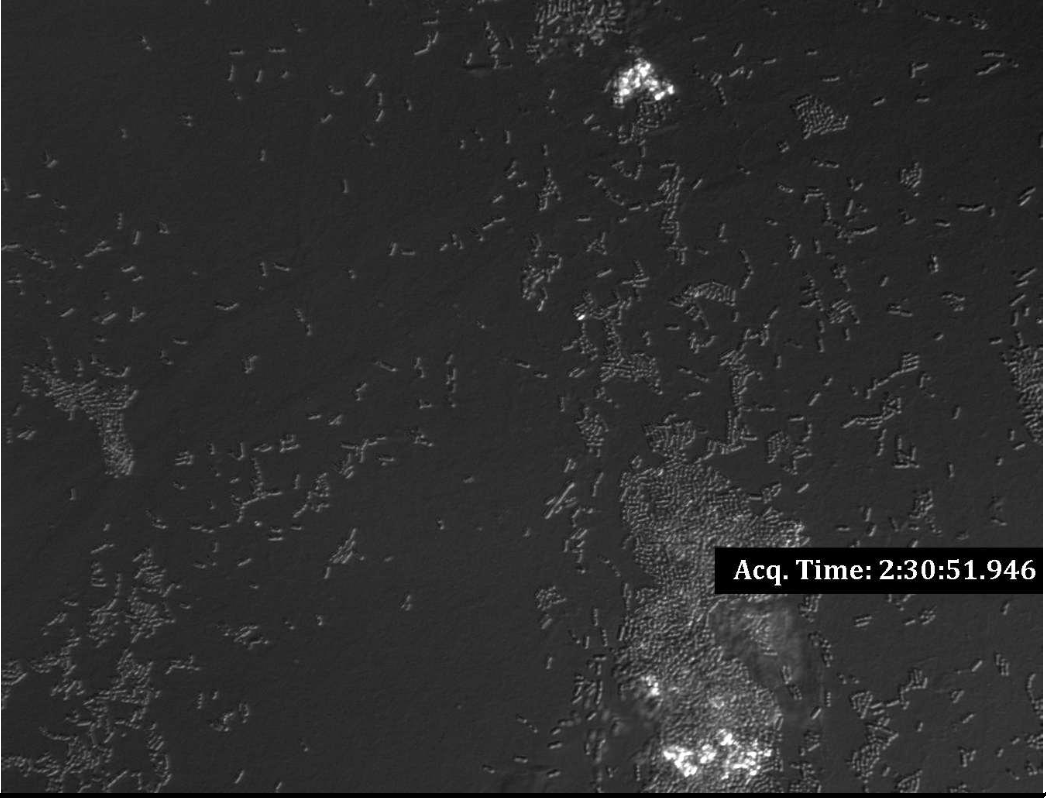

Supplement: File S2 — Microscopy images. Isolation of type I and type II persister cells. As described in Figure 6, time-lapse microscopy was implemented to validate the isolation of type I and type II persisters using our protocol. Figures S2 to S7 are a sequence of images taken of the isolation of type I persisters from a stationary phase culture of hipA7 (TH1269) whilst Figures S8 to S12 correspond to the isolation of type I and type II persister cells from an stationary culture of E. coli DS1 (hipQ). (ZIP) [file pone.0088660.s002.zip › PHOTOS/S5.tif]

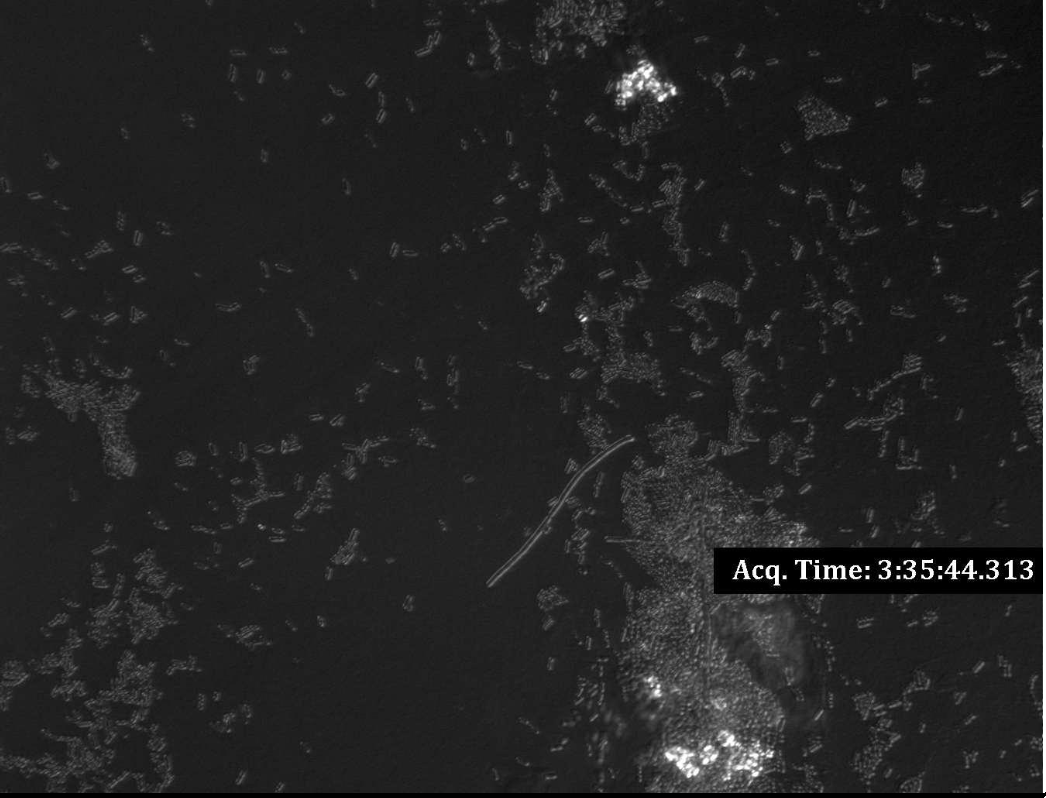

Supplement: File S2 — Microscopy images. Isolation of type I and type II persister cells. As described in Figure 6, time-lapse microscopy was implemented to validate the isolation of type I and type II persisters using our protocol. Figures S2 to S7 are a sequence of images taken of the isolation of type I persisters from a stationary phase culture of hipA7 (TH1269) whilst Figures S8 to S12 correspond to the isolation of type I and type II persister cells from an stationary culture of E. coli DS1 (hipQ). (ZIP) [file pone.0088660.s002.zip › PHOTOS/S6.tif]

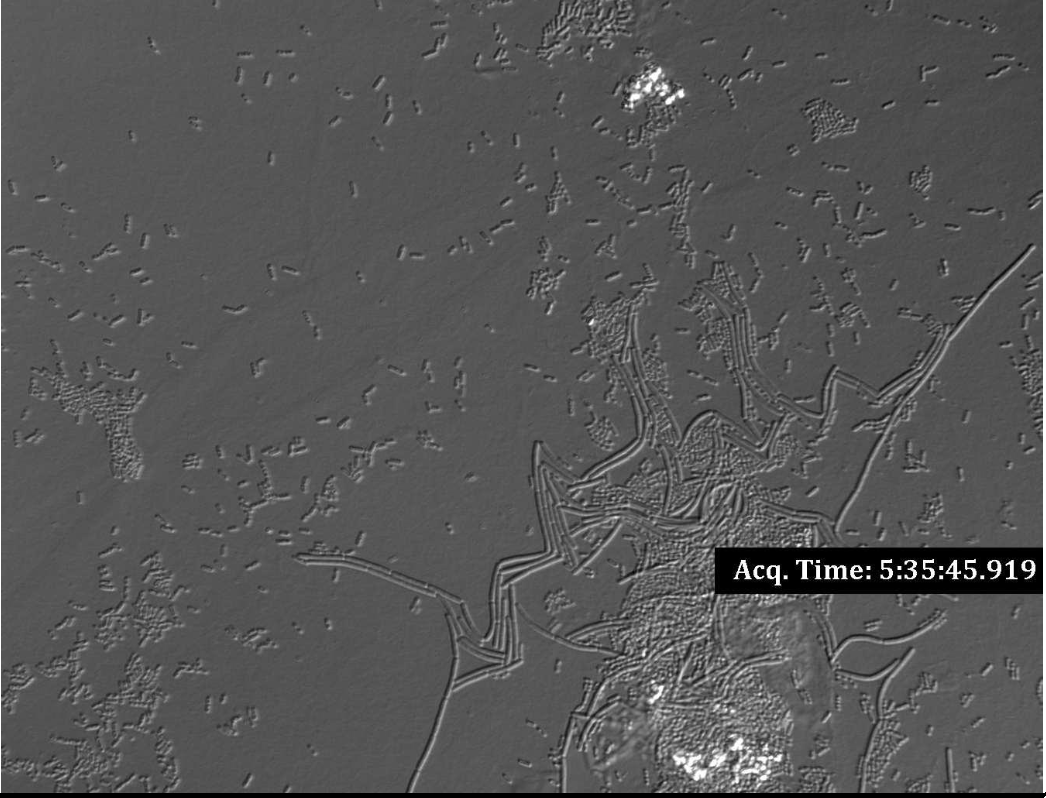

Supplement: File S2 — Microscopy images. Isolation of type I and type II persister cells. As described in Figure 6, time-lapse microscopy was implemented to validate the isolation of type I and type II persisters using our protocol. Figures S2 to S7 are a sequence of images taken of the isolation of type I persisters from a stationary phase culture of hipA7 (TH1269) whilst Figures S8 to S12 correspond to the isolation of type I and type II persister cells from an stationary culture of E. coli DS1 (hipQ). (ZIP) [file pone.0088660.s002.zip › PHOTOS/S7.tif]

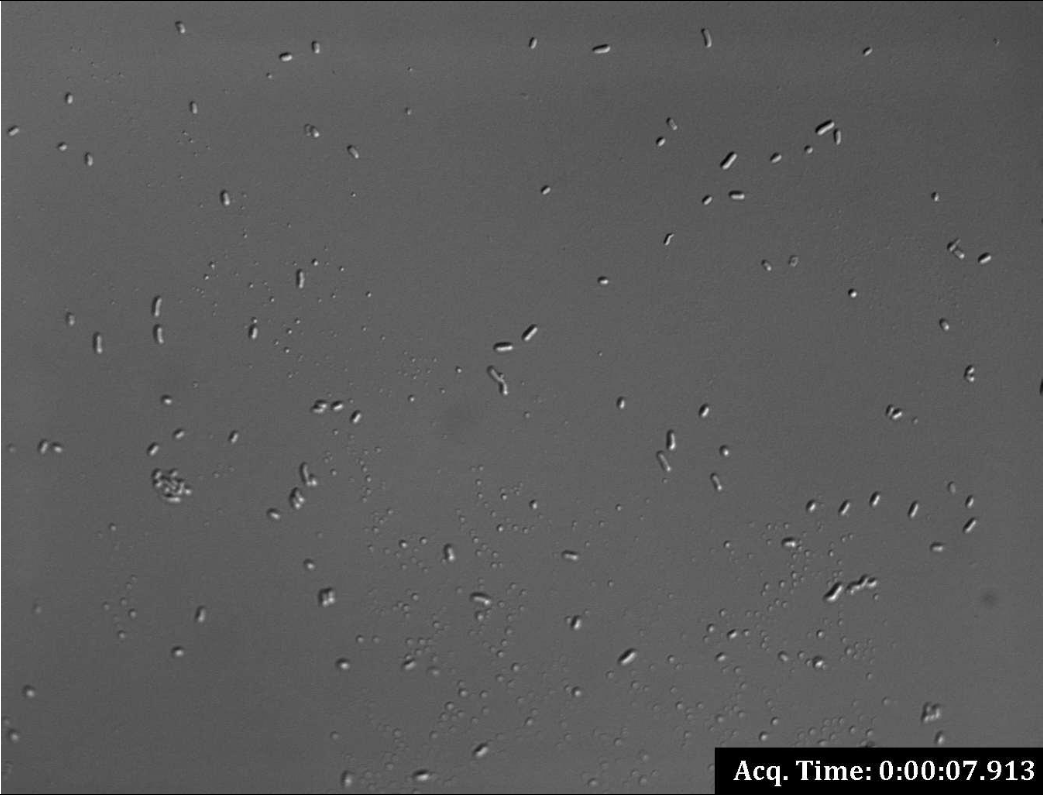

Supplement: File S2 — Microscopy images. Isolation of type I and type II persister cells. As described in Figure 6, time-lapse microscopy was implemented to validate the isolation of type I and type II persisters using our protocol. Figures S2 to S7 are a sequence of images taken of the isolation of type I persisters from a stationary phase culture of hipA7 (TH1269) whilst Figures S8 to S12 correspond to the isolation of type I and type II persister cells from an stationary culture of E. coli DS1 (hipQ). (ZIP) [file pone.0088660.s002.zip › PHOTOS/S8.tif]

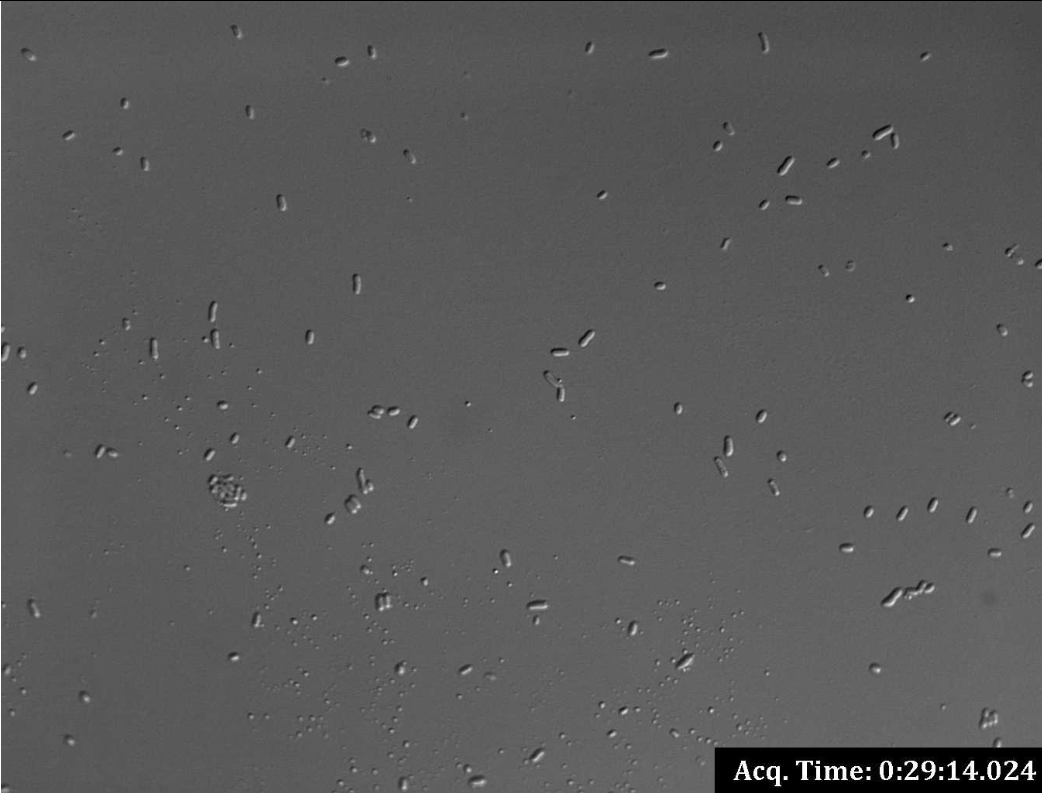

Supplement: File S2 — Microscopy images. Isolation of type I and type II persister cells. As described in Figure 6, time-lapse microscopy was implemented to validate the isolation of type I and type II persisters using our protocol. Figures S2 to S7 are a sequence of images taken of the isolation of type I persisters from a stationary phase culture of hipA7 (TH1269) whilst Figures S8 to S12 correspond to the isolation of type I and type II persister cells from an stationary culture of E. coli DS1 (hipQ). (ZIP) [file pone.0088660.s002.zip › PHOTOS/S9.tif]
